# Avulsion Fracture of the Posterior Tibiofibular Syndesmosis

**DOI:** 10.5334/jbsr.2881

**Published:** 2022-09-21

**Authors:** Wouter Schroven, Peter Boone, Filip Vanhoenacker

**Affiliations:** 1 AZ Sint-Maarten Mechelen and University Hospitals Leuven, BE; 2 AZ Sint-Maarten Mechelen, BE; 3 AZ Sint-Maarten and University (Hospital) Antwerp/Ghent, BE

**Keywords:** PITFL, AITFL, syndesmosis injury, tibial lip fracture

## Abstract

**Teaching Point:** A posterior tibial lip fracture is a rare avulsion fracture at the tibial insertion of the posterior tibiofibular ligament that causes significant ankle instability and often requires surgical intervention.

## Case History

**Figure 1:**
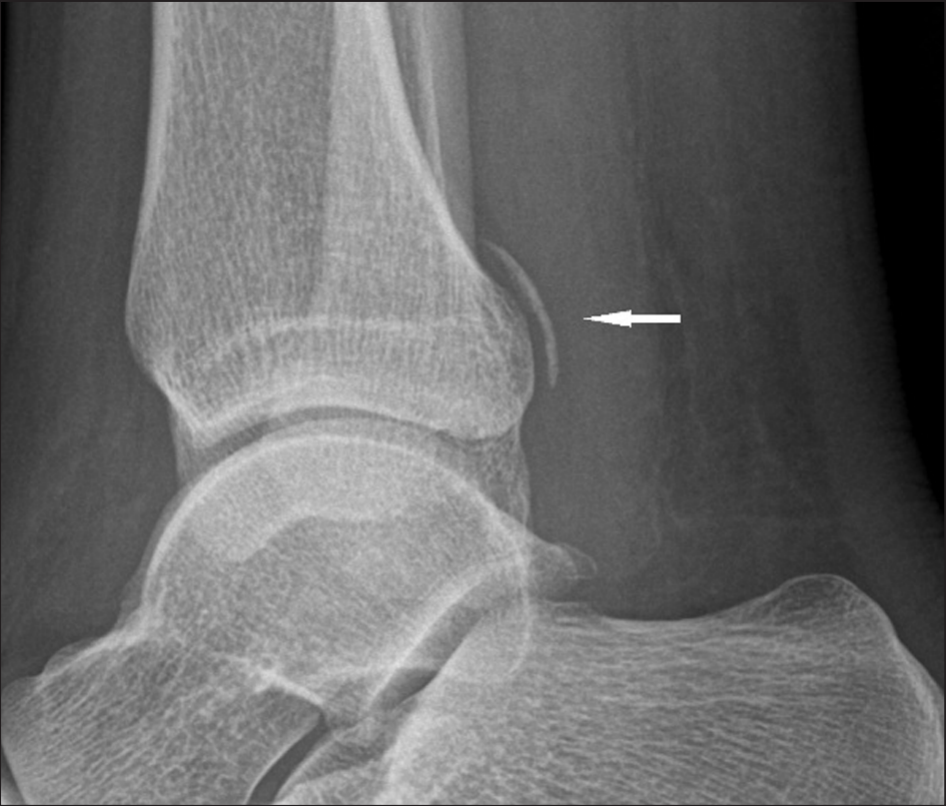


**Figure 2:**
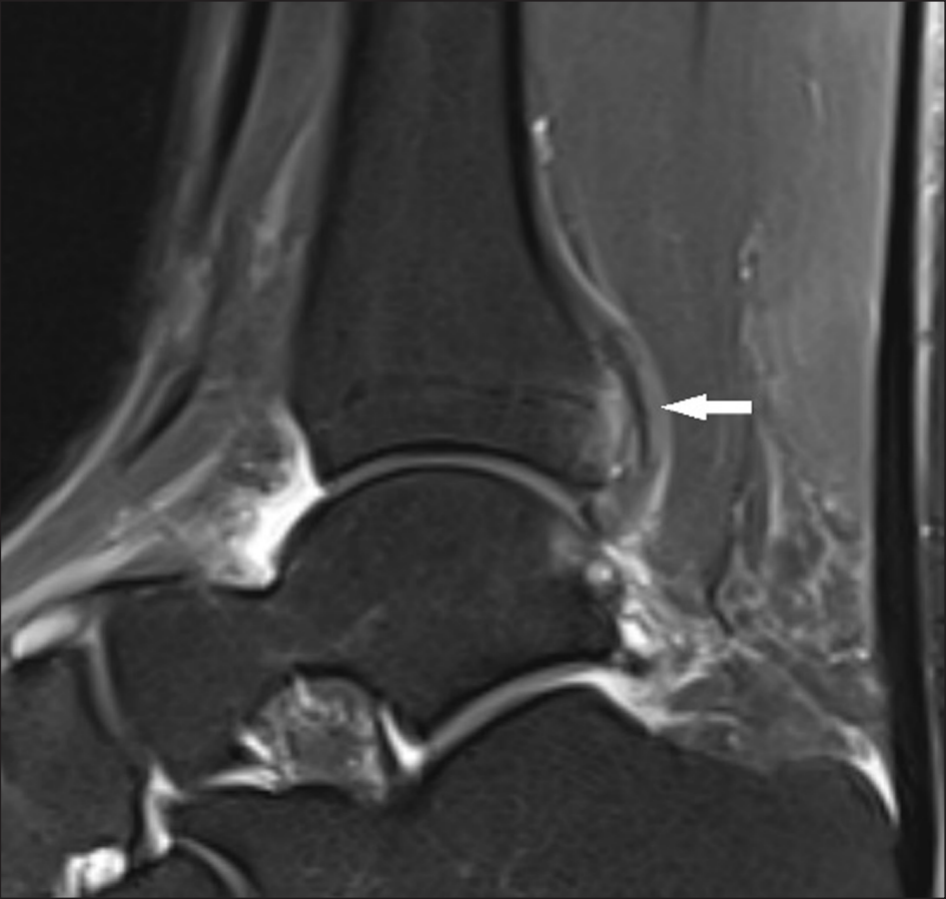


A 41-year-old male presented with pain in the right calf after a blunt trauma on his leg. Conventional radiography revealed a proximal fibula fracture and a bone fragment posterior to the distal tibia (Figure 1) were seen. Due to persisting pain at the ankle after conservative treatment, magnetic resonance imaging (MRI) of the ankle was performed. MRI showed a grade 3 sprain of the anterior tibiofibular ligament (AITFL) and bone marrow edema of the posterior malleolus at the attachment of the posterior tibiofibular ligament (PITFL) along with a posterior tibial lip fracture (PTLF) (Figure 2).

**Figure 3:**
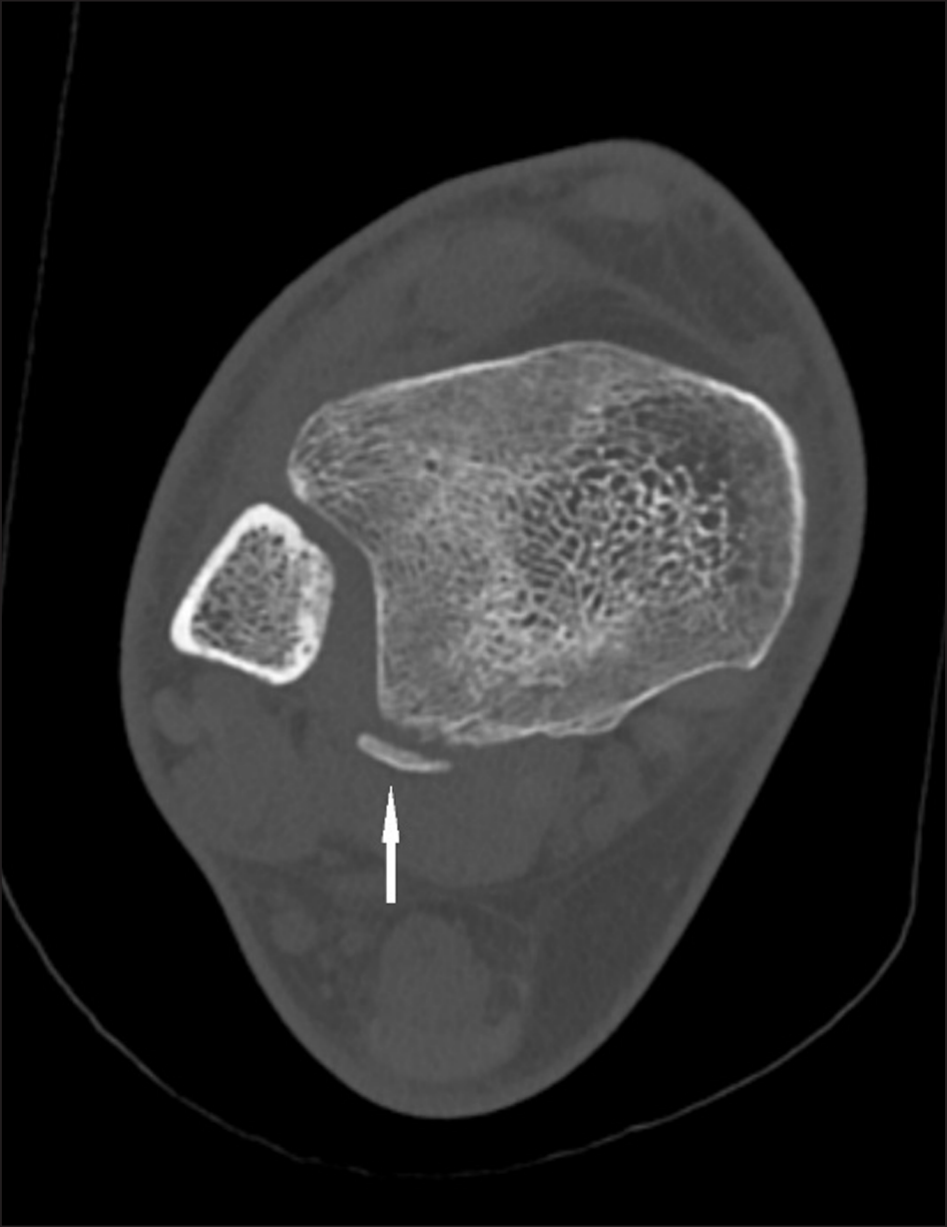


Subsequent CT-examination confirmed the diagnosis of a PTLF with curvilinear morphology (Figure 3).

## Comment

Syndesmosis injuries occur typically when the ankle is externally rotated and dorsiflexed. At initial presentation, diagnosis is missed in more than 20% of cases, particularly if the PTLF is not correctly interpreted as a sign of a significant avulsion fracture of the PITFL. In the latter scenario, patients present with persistent pain on walking, increased stiffness on dorsiflexion and instability. PITFL tears are always accompanied by an AITFL tear. An AITFL tear is accompanied by a various degree of PITFL tear. Injuries to the PITFL are rare since it is the strongest ligament of the syndesmosis, providing 41% of stability [1]. Excessive stress is more likely to lead to an avulsion fracture rather than a rupture of the ligament [1].

Therefore, radiographs should be scrutinized for the presence of a PTLF. If present, patients should be referred for subsequent MRI of the ankle.

On MRI the PTIFL is normally hypointense on both T1 and T2 imaging. Partial tears are characterized by fluid signals transecting the ligament fibers or a hyperintense signal of the ligament. Complete tears show a complete discontinuity of the ligament fibers. Acute and chronic tears are distinguished from each other, respectively, by the presence or absence of edema.

Ultrasound (US) also has proven to be effective in diagnosing syndesmosis injuries with high inter- and intra-observer correlation (>0.8). In PTLFs, US can detect the hyperechoic avulsed fragment, usually with posterior acoustic shadowing associated with surrounding hyperemia in the acute and subacute stages.

Surgical reconstruction is therefore usually indicated to preserve stability [1]. With reduction of the avulsion fracture, the ligament is stabilized.

In conclusion, when there is a history of external rotation and dorsiflexion with a PTLF on radiographs, there should be a high index suspicion for syndesmosis injuries. Both MRI and US offer good results in confirming syndesmosis injuries.
